# Life-threatening cardiac tamponade: a rare complication of acupuncture

**DOI:** 10.1186/1749-8090-9-61

**Published:** 2014-03-31

**Authors:** Kook-Jin Chun, Sang-Gwon Lee, Bong Soo Son, Do Hyung Kim

**Affiliations:** 1 Cardiovascular Center, Pusan National University Yangsan Hospital and Medical Research Institute, Bumuh-ri, Moolgum-up, Yangsan, Gyungsangnam-do 626-770, South Korea; 2 Department of Thoracic and Cardiovascular Surgery, Pusan National University Yangsan Hospital and Medical Research Institute, Yangsan, South Korea

**Keywords:** Acupuncture, Complications, Cardiac tamponade

## Abstract

Acupuncture as an ancient Chinese treatment has proven effective and is utilized worldwide. Although it is generally believed to be a safe clinical procedure, serious lethal complications including death have been reported. We present a rare case of life-threatening cardiac tamponade due to penetration of an acupuncture needle directly into the right ventricle.

## Background

Acupuncture is an ancient form of Chinese medicine involving the insertion of solid filiform acupuncture needles into the skin at specific points on the body to achieve a therapeutic effect. It has become popular in the rest of the world as well as East Asia in recent decades. It is regarded as a safe treatment method for many conditions, if it is performed according to established safety rules at appropriate anatomical regions. A number of large surveys on the safety of acupuncture have been conducted, mainly in Europe. However, serious lethal complications including death have been reported although it is generally believed to be a safe.

We present a rare case of life-threatening cardiac tamponade due to penetration of an acupuncture needle directly into the right ventricle.

## Case presentation

**Figure 1:**
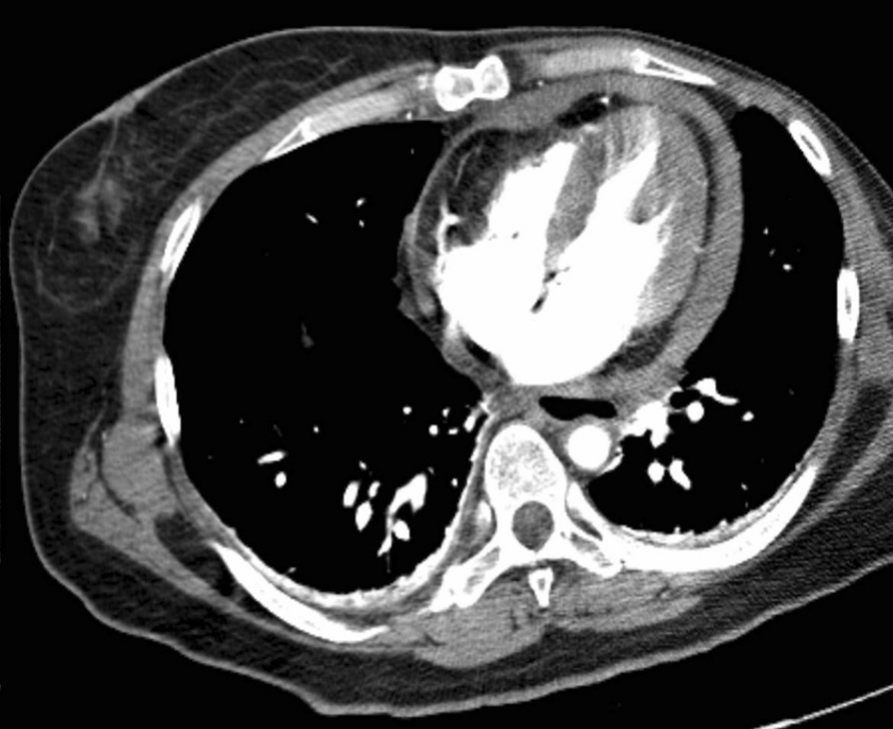
A computed tomogram demonstrated a pericardial fluid collection around the heart.

**Figure 2:**
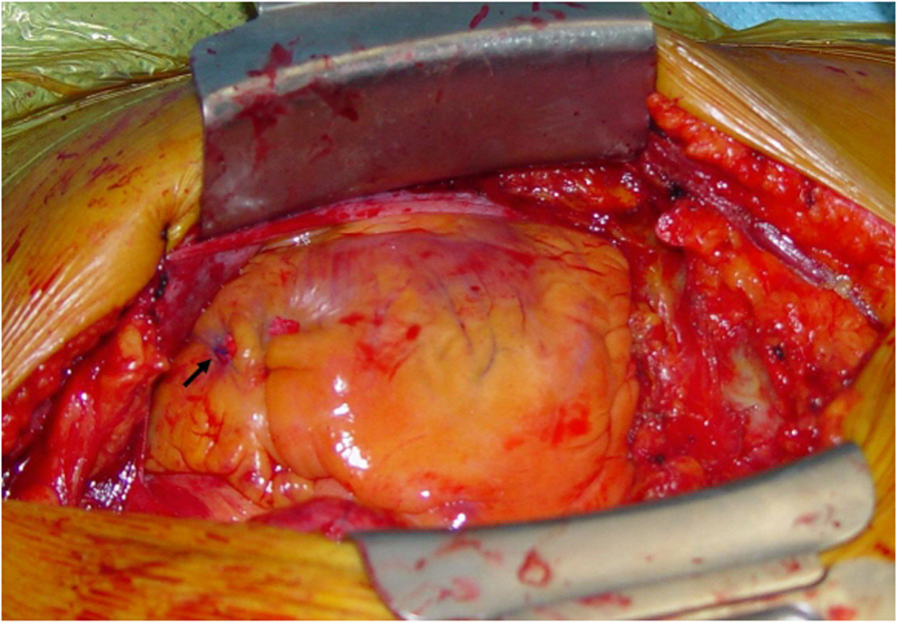
Intraoperative view of the perforating lesion which was sutured (arrow).

A 48-year-old korean woman was brought to the emergency department due to hypotension and drowsiness. She had a history of left mastectomy followed by chemotherapy due to breast cancer seven years ago. Otherwise her past medical history was unremarkable. She suddenly developed bradycardia, syncope and became comatose about 10 minutes after an acupuncture session, in which a needle was inserted into the left anterior parasternal area at the level of the 4^th^ intercostal space by a professional acupuncturist. Immediate cardiopulmonary resuscitation was performed successfully and the patient was transferred to our hospital. On physical examination, she had jugular venous distension with distant heart sound. Systolic blood pressure was 50 mmHg with a heart rate of 120 bpm. After rapid administration of intravenous normal saline with inotropic agents, blood pressure was restored up to 90/60 mmHg. Transthoracic echocardiogram revealed a moderate amount of pericardial effusion with early diastolic collapse of the right atrium which was predominantly placed beyond the left ventricle and a computed tomogram demonstrated a pericardial fluid collection around the heart (Figure 1). Under the presumptive diagnosis of traumatic hemopericardium, the patient underwent emergent pericardial drainage via pericardiocentesis. But emergent cardiac surgery was decided because active bleeding was not stopped. After median sternotomy, a large amount of clotted material was evacuated from the pericardial cavity. A tiny perforating hole (3 mm in diameter) that was continuously oozing blood was noted in the anterior wall of the right ventricular outflow tract (Figure 2). The lesion was directly sutured without the need for cardiopulmonary bypass. The postoperative course was uneventful and she was discharged four days after the operation.

## Discussion

This case highlights that acupuncture, which entails inserting and manipulating fine needles into specific bodily points with the aim of relieving pain and for therapeutic purposes, can cause a catastrophic conditions, including life-threatening cardiac tamponade caused by direct penetration of an acupuncture needle into the right ventricle. Although acupuncture is a very safe procedure in the hands of a competent practitioner, several serious complications have been described in the scientific literature [1]. Thus far, seven cases of cardiac tamponade associated with acupuncture have been reported [1-4]. It is said that direct perforation of myocardium or migrated acupuncture needles from a remote site can cause cardiac tamponade. Because the distance from the surface of the skin to the anterior surface of heart was estimated to be only about 13–19 mm, even an experienced acupuncturist might penetrate the cardiac chamber or the coronary arteries with acupuncture needles (which are 30 mm in length). In this case, the acupuncture needle may have been inserted in a perpendicular direction through a chest wall likely rendered thinner by a mastectomy. Two similar case reports have been published documenting fatal cardiac tamponade caused by direct mechanical injury to the right ventricle in an elderly patient with emaciation and by acupuncture through a congenital sternal foramen [2,4]. A sternal foramen was not found during sternotomy in our patient. Echocardiography is a valuable tool for the evaluation of shock, cardiac tamponade and hemodynamic instability of unknown origin. With the help of echocardiography, we swiftly made an emergent decision that lead to successful management of this case.

## Conclusion

The diagnosis of cardiac tamponade induced by an acupuncture needle should be considered when unexplained shock after an acupuncture procedure at the chest wall is found.

## Consent

Written informed consent was obtained from the patient for publication of this case report and any accompanying images. A copy of the written consent is available for review by the Editor-in-Chief of this journal**.**

## Competing interests

The authors declare that they have no competing interests.

## Authors’ contributions

KJC drafted the manuscript, SSB analyzed and interpreted the patient data, SGL performed the surgery and was a major contributor in writing the manuscript and DHK involved in the crucial revisions of manuscript. All authors read and approved the final manuscript.
